# Is the National Stamina Lowered?

**Published:** 1918-11-30

**Authors:** 


### IS THE NATIONAL STAMINA LOWERED?

Sir John Collie, Director of Medical Service in the Ministry of Pensions, was prevented from reading a paper at the Royal Institute of Public Health on November 20. In his stead Major Basil Rhodes read an abstract of it. According to the *Morning Post* report, "as regards health it was shown by statistics that the effect of the war had been very great on the nation, mainly in the result of loss of stamina brought about by the limitation or deterioration of food."

Statistics can be made to prove anything, and these statistics are not to hand; but the general statement can be criticised. In the first place, what is the comparison? What statistics on national stamina existed before the war with which to compare the war-time statistics? Has account been taken of the fact that of the population about 10 per cent. has been taken into the Army, drilled, trained, and thoroughly well fed? These men are part of the nation. Surely their stamina is not reduced. That it is not is shown by the deeds they have done. If the statistics of national stamina have been concerned with the less virile men who have stayed at home and have disregarded the fighting men, there is at least one obvious fallacy in them.

Has the mass of the population of these islands really had an insufficient quantity of nourishing food? Bread, the staple, the real staff of life, has been artificially kept cheap. Meat, a much less important food, has been so rationed that everyone has been able to have a fair share, and a share per week sufficient for physiological needs. Meat has been dear without question, but wages have risen so much more than the cost of necessaries that probably most wage-earners have been able to buy more meat per week during the war than they bought before it. It is common knowledge that the women have been earning great sums of money. In many cases girls who lived at home and were dependent on the wages of the head of the family have been able not only to relieve that head of all cost of their keep, but to contribute to the common exchequer. Thousands of working-class women with separation allowance and their earnings have been drawing from £100 to £200 a year. The large expenditure on luxuries and amusements of munition workers, miners, taxi-drivers, and a host of other wage-earners has been notorious. Workers have bought millions of pounds' worth of War Savings Certificates. People whose food is less than that necessary to good health and good work do not spend on luxury and amusement money that could satisfy physiological craving. Altogether there does seem to be good reason to doubt if there really has been such deterioration of food as would suffice to lessen stamina. There are other statistics of sorts that prove that, as the result of war-time conditions, national health has improved. It may be that the true case is that because the conditions of war-time have made the health of the nation a matter of great importance, that matter has been more closely considered than heretofore, and so dangers that lay hidden have been brought to light.

Great Britain is an industrial country; the population is an urban population; the great majority lives in large towns, and prefers life in large towns to any other. In the towns are glare and crowds, amusements of an exciting nature, public-houses, bands, billiards, cinema shows, music-halls, theatres, and a thousand other amusements. Very few of this urban population like rural life or have any craving for the peace and simple pleasures of the country. At holiday times the crowds move from the towns where they live their lives and work, to other great towns where they can find the amusements and excitements they love combined with the novelty of the sea—such places as Margate, Blackpool, Brighton. The comparatively speaking few that go into a country place go in parties with brakes and beer, and picnic in crowds where there are swings and shooting-galleries and coconut-shies. They show no other appreciation of natural or cultivated beauties than to break off branches of flowering shrubs or to dig up the primrose roots and ferns, till that countryside which suffers and makes profit from trippers is denuded of some of its chief beauties. The mass is of urban tastes, enjoys urban life, would be bored by life in a sparsely inhabited country district, because it has not learnt to use leisure. It has not the culture that makes a man happy and content with beautiful surroundings, and simple, intellectual pleasures.

To a portion of the British population, after work, quiet, and a book, beautiful scenes and the varied life of bird and beast and plant are the greatest pleasures, but power to appreciate such joys implies a certain heredity, and a cultivation of the mind of the kind yet to be given by national school teachers in national schools. The factory for the working hours, and music-halls and picture-palaces in leisure hours, is not the life that builds up brain, muscle, or constitution. There is not the least likelihood that the urban populations will change their taste for a town life to a taste for a country life, and, though the conditions under which they live the unhealthy life of towns may be made more healthy, yet it is doubtful if life in large towns can now be such as to evolve a healthy population.

It has been stated that emigration from the country keeps the towns full, but that the progeny of the emigrants does not survive beyond the fourth generation. The great-grandchildren of the healthy country emigrant, without stamina and sterile, cease to exist and leave no descendants. His children and his grandchildren have deteriorated till the end of his line has come. It seems, therefore, that the wise policy is so to arrange matters that there may be every inducement to our comparatively small number of country folk to increase and multiply so that our towns may be fed with their fresh and vigorous blood and the inevitable wastage of town life fully supplied. The real remedy is to teach our children to enjoy a simpler, healthier life than the life now lived in towns, and to find happiness in intellectual resources, by the exercise of their own physical and mental gifts. Continuous organised effort should wean them from the craving for pleasure that is not always happiness and from exciting amusements which are not conducive to the healthiness and vigour of their race.

